# Autonomous Tool for Monitoring Multi-Morbidity Health Conditions in UAE and India

**DOI:** 10.3389/frai.2022.865792

**Published:** 2022-04-28

**Authors:** Shadi Atalla, Saad Ali Amin, M. V. Manoj Kumar, Nanda Kumar Bidare Sastry, Wathiq Mansoor, Ananth Rao

**Affiliations:** ^1^College of Engineering and Information Technology, University of Dubai, Dubai, United Arab Emirates; ^2^Department of Information Science and Engineering, Nitte Meenakshi Institute of Technology, Bangalore, India; ^3^Ramaiah Medical College and Hospitals, Bangalore, India

**Keywords:** multi-morbidity, health, old age patients, autonomous tools, real-time

## Abstract

Multi-morbidity is the presence of two or more long-term health conditions, including defined physical or mental health conditions, such as diabetes or schizophrenia. One of the regular and critical health cases is an elderly person with a multi-morbid health condition and special complications who lives alone. These patients are typically not familiar with advanced Information and Communications Technology (ICT), but they are comfortable using smart devices such as wearable watches and mobile phones. The use of ICT improves medical quality, promotes patient security and data security, lowers operational and administrative costs, and gives the people in charge to make informed decisions. Additionally, the use of ICT in healthcare practices greatly reduces human errors, enhances clinical outcomes, ramps up care coordination, boosts practice efficiencies, and helps in collecting data over time. The proposed research concept provides a natural technique to implement preventive health care innovative solutions since several health sensors are embedded in devices that autonomously monitor the patients' health conditions in real-time. This enhances the elder's limited ability to predict and respond to critical health situations. Autonomous monitoring can alert doctors and patients themselves of unexpected health conditions. Real-time monitoring, modeling, and predicting health conditions can trigger swift responses by doctors and health officials in case of emergencies. This study will use data science to stimulate discoveries and breakthroughs in the United Arab Emirates (UAE) and India, which will then be reproduced in other world areas to create major gains in health for people, communities, and populations.

## Introduction

Researchers have collected massive amounts of data thanks to recent technological advancements throughout the world. Data availability and quality are important factors affecting our ability to improve individual and community health, from delivering treatment to performing scientific research, from rural clinics to the most modern genomics facilities. The capacity to completely extract relevant insights from this data will lead to faster discoveries and inventions that will positively influence health in the United Arab Emirates (UAE), India, and probably the rest of the world in due course. Rapid advances in data science, such as new methods to describe, collect, store, integrate, and analyze large scale, structured and unstructured heterogeneous data sets, as well as new data modeling methods such as artificial intelligence, machine learning, advanced deep learning, digital phenotypes, and three-dimensional imaging, are expected to have a high impact on the outcomes of behavioral and biomedical research leading to health improvement for populations and individuals, over the next decade. Traditional and publicly available datasets (e.g., surveillance, national health systems, and surveys) are becoming richer and deeper, while new other sources of datasets generated by new technologies and wearable sensors [e.g., social media streams, smartphones, global positioning system (GPS) data, wearables devices, electronic medical records datasets, genomics data, and bio-imaging] are emerging and being intensively explored for new opportunities and findings. Advances in diagnostics, technological development, and the possibility for public health are all based on progress in the generation of massive new data sets and sophisticated ways for mining hidden and interesting patterns of these datasets (Marston et al., 2019; Abuelkhail et al., 2021; Majnarić et al., 2021; Poongodi et al., 2021).

This study concept will use data science to stimulate discoveries and breakthroughs in the UAE and India, which will then be reproduced in other world areas to create major gains in health for people, communities, and populations. The multidisciplinary area of inquiry in which quantitative and analytical tools, procedures, and systems are developed and deployed to extract information and insights from increasingly massive and/or complicated quan1tities of data is described as data science in this study. Autonomous tool for monitoring multi-morbidity health conditions improves medical quality, promotes patient security and data security, lowers operational and administrative costs, and provides insights to the people in charge to make informed decisions.

Decades of infrastructure development and training in the UAE and India have created attractive research prospects to address the UAE and India's disproportionate part of the global illness burden. Data science has the potential to have a substantial influence on both qualitative and quantitative research and health in the UAE and India. New relevant, economical, acceptable, and scalable solutions may be produced by exploiting the current digital infrastructure. For example, in the UAE and India, widespread mobile phone service has resulted in significant advancements in banking, logistics, immigration, and other industries, including agriculture. It can also quickly develop healthcare delivery systems by bringing the clinic to the patient *via* point-of-care technology and self-management systems, with applications to rural and underserved communities worldwide. Also, as per UNESCO Human Development Index (HDI), UAE is considered very highly developed with a ranking being 31, while India is considered low with a ranking of 131. Contrasting best practices in these two extreme conditions facilitate broader learning and application from the proposed concepts. Following are the facets/applications of the proposed conceptual model:

Promote patient-centered healthcare at a lesser cost. Enhance the quality of care and information sharing among medical personnel.

- Educate health workers and patients through training.
- Encourage patients and healthcare providers to create new kinds of humane relationships.
- Cut down travel time and receive remote consultation, diagnosis, and treatment from specialists in far-flung facilities through telemedicine.
- Monitor public health threats.
- Promote patients' self-diagnosis or monitor illnesses.

## Increasing ICT Usage Trends in the Elderly Population Group

**Figure 1:**
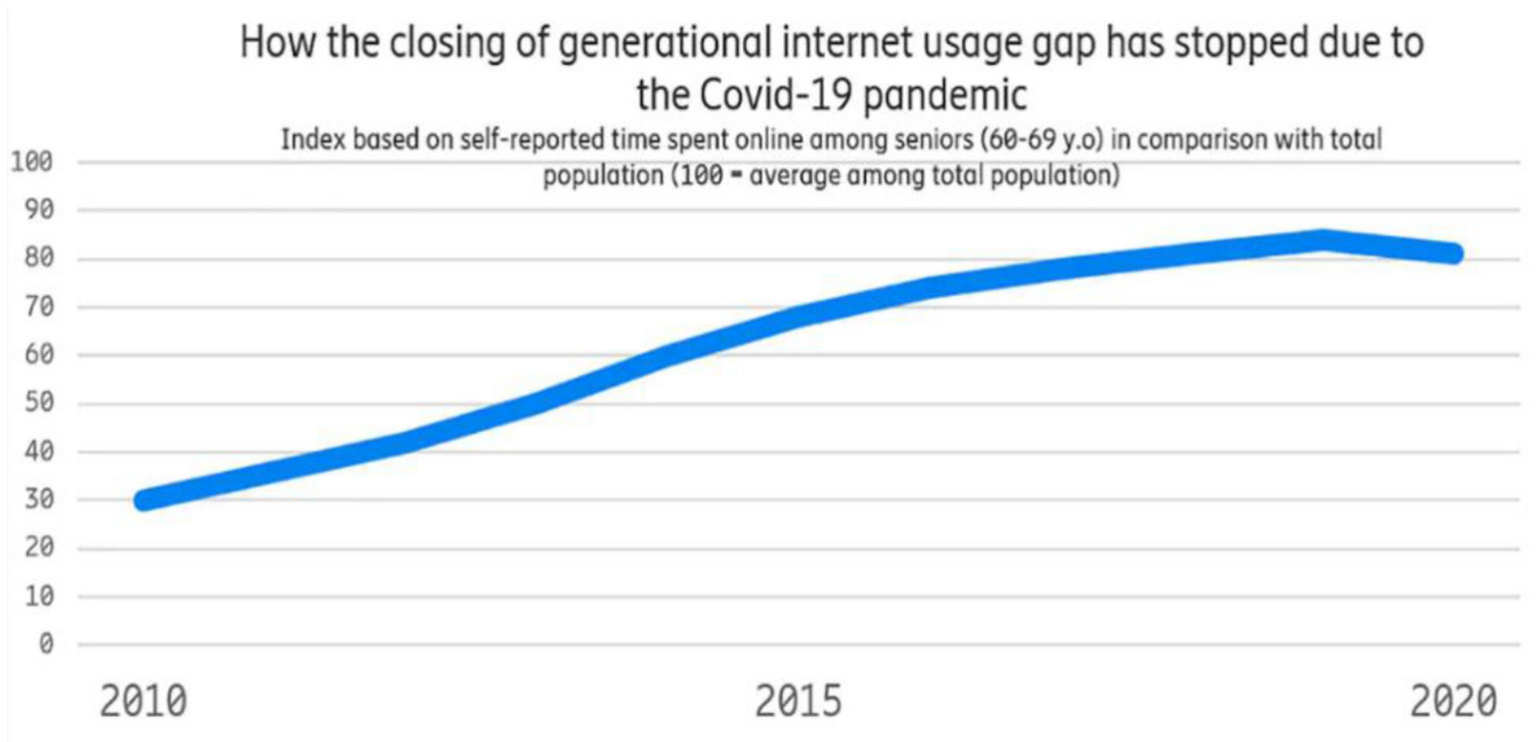
How the internet usage gap has stopped since the outbreak of the pandemic.

Figure 1^1^ shows how seniors aged 60–69 years old had been closing the digital gap (in the sense of time spent online) compared to the total population.

One of the reasons that seniors have been catching up with younger generations is that each year, newer, more technologically savvy seniors replace older seniors who have reached the top limit of the age interval. Another explanation is that “new” seniors are more likely to find it simpler to adopt new internet habits once they reach retirement age. This is owing to their relatively high level of digital expertise, but it is also frequently due to family and friends who encourage them and want them to benefit from gadgets and applications for purposes such as communication, knowledge, or simply having fun.

However, as the COVID-19 crisis erupted, the generational digital divide ceased to close. Not because seniors used the internet less—in fact, they increased their use. The use of new technologies by the senior population greatly helps to a higher quality of life by increasing indices of everyday living such as transportation, communication, and social involvement. The older population is increasingly using modern technology than before. It is found that 77% of those over 65 years used the internet at home in 2020 (Figure 1). Similarly, it has been recorded that access to Internet/online services by old-aged women has doubled since 2011 (Marston et al., 2019).

**Figure 2:**
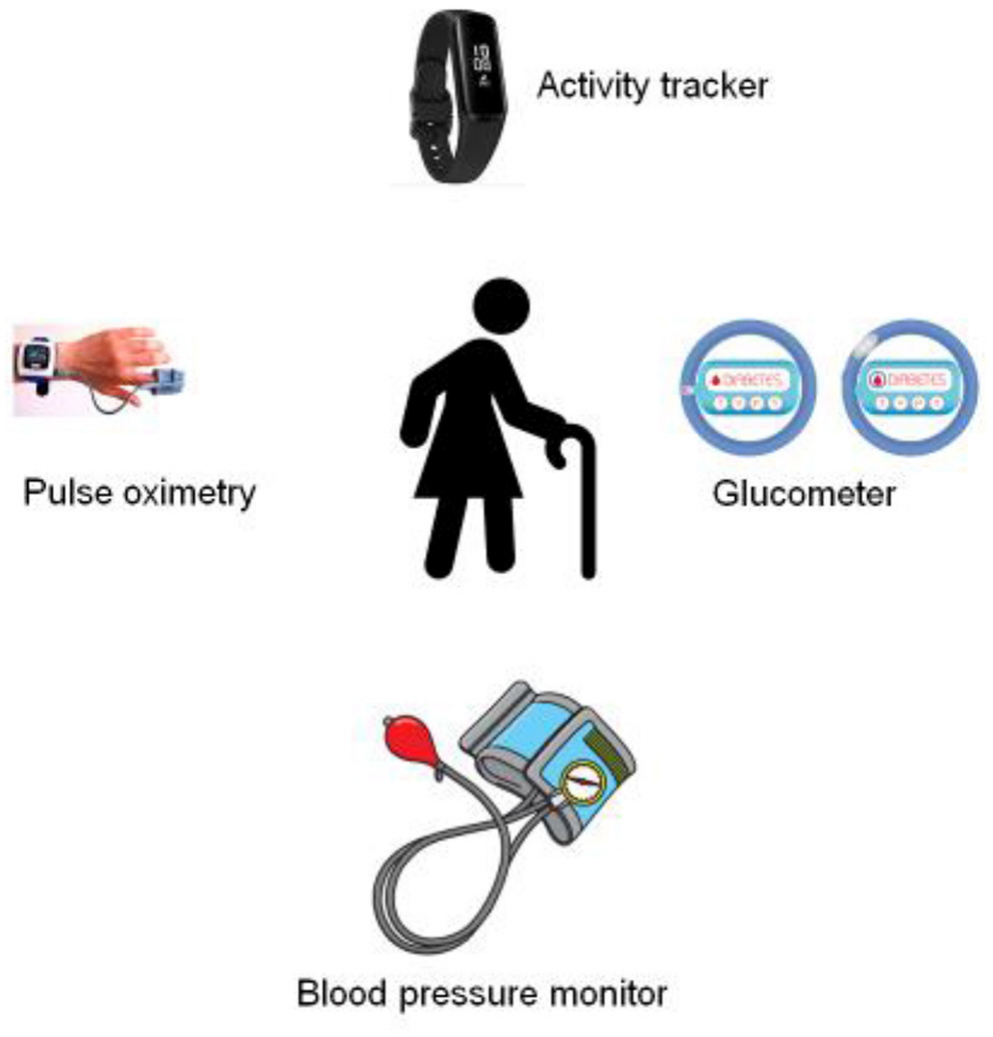
Wearable sensor networks.

Exploring the appropriate statistical variables and models for developing the machine learning component, which has been proposed in this study (**Figure 5**), extends findings from relevant prior research. The training phase of the model collects data variables primarily based on publicly available health conditions datasets. These variables include data about blood pressure, pulse oximetry, the concentration of glucose in the blood, activity tracking, sleep tracking with the corresponding prediction class; in addition to patient feedback and evaluation data (Dinsmore et al., 2021); finally, data related to health trajectories from Healthcare Administrative Databases (HADs) such as diagnoses and medication prescriptions are also included (Veronica et al., 2022). The production phase of the model collects data coming from the proposed tool and its associated sensory fabrics, as described in Figures 2, **4** to predict emergent health situations requiring interventions such as early hospital admission, diagnosis, clinical procedures, and medications, will be compared with deep learning (DL) and convolutional neural network (CNN) (Nguyen et al., 2016; Pham et al., 2016) vs. traditional machine learning algorithms, e.g., Bayesian probabilistic model, k nearest neighbors, logistic regression, support vector machines, and decision tree (Deparis et al., 2018; Hansen et al., 2018; Khalid et al., 2018; Noh et al., 2019; Ben-Assuli and Padman, 2020; Franz et al., 2020; Veronica et al., 2022).

Notwithstanding, these traditional techniques suffers from multiple issues in real-time applications necessary in health application as they rely on feature selection and generation. Moreover, their performance decreases on high-dimensional datasets or large-scale datasets. To overcome the limitation of traditional methods, deep learning-based methods such as the convolutional neural network (CNN) model and long short-term memory (LSTM) models will be alternatively investigated.

The performance of the proposed machine learning algorithms will be assessed through relevant prediction score metrics, such as accuracy, F1-score, precision, and recall. The performance scalability of each algorithm will be evaluated based on the prediction latency time and system requirements in terms of processing and memory units. However, the proposed method will extend prior research in two specific innovative areas: first, assess the scalability performances of a holistic, big data framework to support the integration of several health applications to address the complex care needs of patients with multi-morbidity. Second, assess the performance of traditional and deep learning machine algorithms against several prediction accuracies and the production setup suitability for real-time healthcare applications targeting patients with multi-morbidity.

### Research Scope

This research concept provides a natural technique^2^ to implement a smart preventive health care solution. The proposed solution improves safer and more efficient patient care, such as elder persons with multi-morbidity diseases. The purpose is to enhance the elder's limited ability to watch, predict and respond to critical health situations. Autonomous monitoring of the patient's health status and alert doctors and patients themselves helps lower unexpected health complications. The following four objectives help in achieving the research concept scope:

**Objective-1: To redefine commodity technology requirements to monitor the health conditions of patients with multi-morbidity in real-time**

- This research leverages smart devices such as wearable sensors and mobile phones for monitoring elderly persons with a multi-morbid health conditions that will work seamlessly anytime, anyplace for anyone. To achieve this objective, the specifications, functionalities, application, and technology requirements shall be identified and investigated to address the complex needs of elderly patients. This objective leverages the co-design approach (Spinuzzi, 2005) to involve all stakeholders to gather and the MoSCoW method (Clegg et al., 1994) to prioritize the system requirements.

**Objective-2: To build a platform of autonomous health data collection and processing that will allow smooth interactions between patients, his/her mobile App, and the back-end big data platform**

- Given the heterogeneity, ubiquity, and increasing autonomy of smart commodity devices, we foresee the need for a powerful mobile app and data management infrastructure specially designed to collect health information from thousands of diverse sources, providing their data in a variety of formats, granularities, and at different speeds, e.g., persons who suffer from two or more chronic diseases simultaneously. We also foresee the need to process these huge data streams in real-time. The system will therefore have to process directly as soon it is received instead of using the typical store-and-process paradigm (Sharma et al., 2021). The need to extract attributes from those data streams becomes extremely important for meaningful, higher-level information immediately accessible by Apps. The system shall provide users with simple and abstract APIs to access health data generated by devices without dealing separately with the underlying complexity of how data has been collected and data sources are connected to the system. In addition, the infrastructure shall provide a subscription/notification mechanism to notify events that are relevant to the subscribed systems/applications. Correlating and aggregating data sources would be a critical factor in detecting relevant pieces of key health information (Prisacaru et al., 2017).

**Objective-3: To apply machine learning algorithms for predicting and responding to any critical health situations**

- Once the health data are collected, the research proposes to customize the machine learning (ML) algorithms by applying a refined set of parameters to extract essential features that could be fed to ML models with the ability to predict and respond to any critical health situations with autonomous monitoring to alert doctors as well as patients of their unexpected health conditions.
- The results of the ML models are interpreted with the help of appropriate explainable artificial intelligence (AI) techniques.

**Objective-4: To investigate the clinical validity of the holistic platform and its application performance scalability**

- Results from Objective-2 and Objective-3 are expected to give more concise and precise accuracy in predicting and monitoring variables of the patient health conditions. We will observe effects between variables in our preliminary model that concur with the medical literature. Further developments would also include performance analysis of the method for a larger network, the inclusion of the temporal dimension, and different sampling rates per variable.

### Significance

The proposed project would investigate and identify the best ways to integrate wearable sensors and mobile applications to observe the health condition of elderly people with multi-morbidity, including chronic heart failure, diabetes, and chronic obstructive pulmonary disease. The project provides intelligent and integrated platforms leveraging Wearable devices and sensors technologies to address the complex care needs of patients with multi-morbidity through monitoring the patients' health conditions in real-time. Moreover, applying data processing algorithms to the collected data for building predictive models to identify patients is likely to benefit from these models' recommendations and the timely interventions required. The study findings can significantly impact the way multi-morbidity health conditions are currently diagnosed in the UAE and India in terms of accuracy and robustness.

In addition, reducing human errors, enhancing clinical outcomes, increasing care coordination, boosting practice efficiencies, and collecting data over time are just some of the ways that the proposed conceptual framework can help improve and revolutionize healthcare.

## Literature Review

Commodity devices such as wearable smartwatches and mobile phones have been widely investigated in several health monitoring applications as well as in context-aware situations complementing the elder's limited ability, i.e., rhinitis and asthma, heart rate, blood sugar levels, human body temperature, and fall detection enabled applications (Tarapiah et al., 2017; Bousquet et al., 2018; Mehmood et al., 2019). The integration of wearable and medical devices within home Internet of Things (IoT) based solutions were proposed by the European project entitled ProACT (Integrated Technology Systems for ProACTive Patient-Centered Care). ProACT described methods to observe the health condition of elderly people with multi-morbidity, including diabetes, chronic heart failure, and chronic obstructive pulmonary disease (Dinsmore et al., 2018; Malavasi et al., 2019; Adeniyi et al., 2021). However, very few researchers have been examining how to assess the scalability performances of integrating several health applications into a single device to address the complex care needs of patients with multi-morbidity (Dinsmore et al., 2018; Waschkau et al., 2019). Further, few authors have proposed several data platforms leveraging big data technology that enable the discovery of emergent multi-morbid patterns and emanating longitudinal risk (Malecki et al., 2020; Kishor and Chakraborty, 2021).

Artificial intelligence algorithms such as classification, clustering analysis, and deep learning have been used to build machine learning predictive models to identify multi-morbid patients likely to benefit from these models' recommendations as well as for timely interventions required, for example, facilitate identification of patients having multi-morbidity conditions, disease diagnosis, and prediction system. The Bayesian network probabilistic method was proposed to model patients with multi-morbidity. The authors used features from patient vital signal measurements and activity tracking features. The training dataset was based on publicly available datasets from Irish elderly people, named TILDA (Deparis et al., 2018; Kishor and Chakraborty, 2021). Monitoring multi-morbidity conditions in real-time for thousands of patients simultaneously would generate many data streams. Consolidating these streams into a shared data platform poses the need for assessing the scalable performance of these platforms. However, the authors keep the doors open and pointed necessity to increase the prediction accuracy and to confirm the possibility of safely identifying the non-multi-morbidity patients from the multi-morbidity patients with confidence (Khalid et al., 2018; Noh et al., 2019; Franz et al., 2020).

## Conceptual Framework

**Figure 3:**
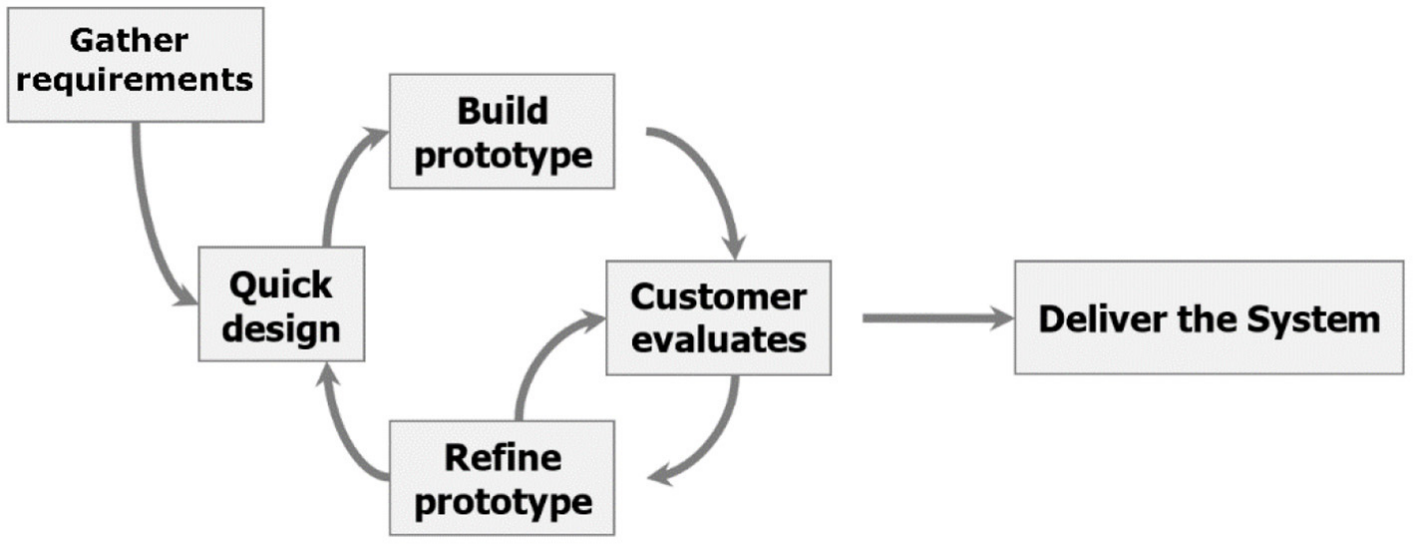
Evolutionary prototyping methodology.

Figure 3 displays the conceptual framework for addressing the research aims and objectives (mainly, objectives 1, 2, and 4). The main development architecture that will be used is prototyping methodology which involves designing and building the system in an agile and patient-centric way.

The standard systems development life cycle (SDLC) system development technique would be utilized to generate the project's final product. In addition, the SDLC will be supplemented by the evolutionary prototyping technique. This entails developing and building a functional scaled-down version of the system in a reliable and organized manner, then refining it through several stages until the final system is obtained. The system analyst would collaborate with the users to establish the fundamental needs. After that, the analyst creates a fast prototype and sends it to the user for feedback. The analyst refines the prototype based on the feedback and delivers the updated version back to the users. This process is repeated until the users are satisfied with the final output.

Improved user participation helps users submit more precise and explicit specifications and capture requirements in concrete form. The fact that the client is testing the prototype helps to keep the developer and client on the same page, improving understanding and communication. Increased engagement raises the possibility of delivering a final solution that will satisfy the stakeholders because the end-user is the most informed person in the problem domain. Furthermore, evolutionary prototyping suggests that the development team may misunderstand many requirements and that only well-understood requirements should be built. This reduces the danger of generating poorly understood components and encourages developers to focus on the parts they are familiar with.

The client can make suggestions for improvements and request adjustments after obtaining the partial prototype. In addition, prototyping saves time and money. Developers can recognize what the end-users want early on, resulting in speedier development and less expensive software.

Overall, the resulting evolutionary engineering, specification, and design methodology comply with the following broad template for each objective iteration: (1) Specification of functionalities for innovation and requirements for sustainable applications. (2) Requirements/innovation engineering and harmonization. (3) Architecture design specification and refinement. (4) Enabling technologies research to implement the architecture and development in short/agile release cycles. (5) Prototype development of the platform, system integration, and testing. (6) Evaluation of the development platform in real application development.

### Key Performance Indicators of the Proposed Conceptual Model

A key performance indicator (KPI) is a quantifiable performance measure over time for a specific strategic objective. KPIs provide targets for teams to shoot for, milestones to gauge progress, and insights that help people make better decisions. Table 1 summarizes the key performance indicators of the proposed conceptual tool.

**Table 1:** KPIs for autonomous tool for monitoring multi-morbidity health conditions in UAE and India.

**On-time completion %**	**Resource capacity %**	**Number of errors**
Milestones on time%	Budget variance (planned vs. actual)	Customer complaints
Estimate to project completion	Budget iterations	Change requests
Adjustments to schedule	Planned value	Billable utilization
Planned vs. actual hours	Net promoter score	Return on investment (ROI)

## Methodology/Approach

The proposed methodology incorporates the principle of co-design, MoSCoW method, user experience, and human–computer interaction. A key aspect often underestimated is the human user interacting with the system. The proposed systems interact with humans to support their daily activities and increase their quality of life. The newest challenge seems to be incorporating human behavior as part of the system itself. The system will deal with large users locally, enabling interaction through dedicated context-aware HMI (human–machine interaction) dynamically reacting to the available system features.

### Objective 1 Is Proposed to Be Achieved as Below

This objective aims to identify case scenarios and business models, including the stakeholders, end-user needs, and (non-) functional requirements for the technologies, products, and value chains, to deliver a new generation of the best ways to integrate wearable sensors and mobile applications to observer the health condition of elderly people with multi-morbidity of critical situations. In addition, the design work in this objective would carry out foresight and feasibility studies to identify architectural quality attributes, system requirements, and reusable core assets that would lead us toward a consolidated system architecture. The purpose is to distinguish a list of generic features for successful long-term deployment of the proposed application and a set of selected features that would be demonstrated in a variety of pilot studies. These pilot studies would be most helpful to analyze the impact of everyday use of our technology in society and identifying new business opportunities.

This objective leverages the co-design approach (Spinuzzi, 2005) to involve all stakeholders to gather and MoSCoW method (Clegg et al., 1994) to prioritize the system requirements.

#### Work Plan Steps

- Define the use cases regarding patient involvement and collect requirements amongst several health service providers by inquiring about external reference entities and literature reviews, workshops, etc.
- Analyze and specify requirements, document, and prioritize their rationale to avoid ambiguities and contradictions, and define the value chain for the described use case and service.
- Utilize the results achieved in the previous steps to define the use cases and their business requirements feeding the technical tasks of the proposal.
- Evaluate the use cases and services in a simulated environment with end-users and gather feedback.

#### Expected Outcomes

- Documentation of the requirements related to leveraging commodity devices such as a wearable smartwatch and a mobile phone app to support patients with multi-morbid health applications, specifically for implementation and demonstration.
- Based on prioritization, list a defined subset of use cases that bring enhanced and true value to the stakeholders through the new platform and system architecture.

### Objective-2 Is Proposed to Be Achieved as Below

This objective aims to demonstrate the key concepts of the project proposal through the development of software platforms with concrete use cases from various components, such as personal sensor networks, Mobile App, and Big Data platforms, through real-life field trials. The objective is to show the proposal's principal innovations in terms of functionality and accuracy of its data capturing, transmission, processing techniques; applicability of the concept in different usage scenarios; easy deployment and seamless integration of the platforms; coexistence, in harmony, of different stakeholders (patient, doctors, health care services, application integrators) thus enabling a successful realization of the concept.

**Figure 4:**
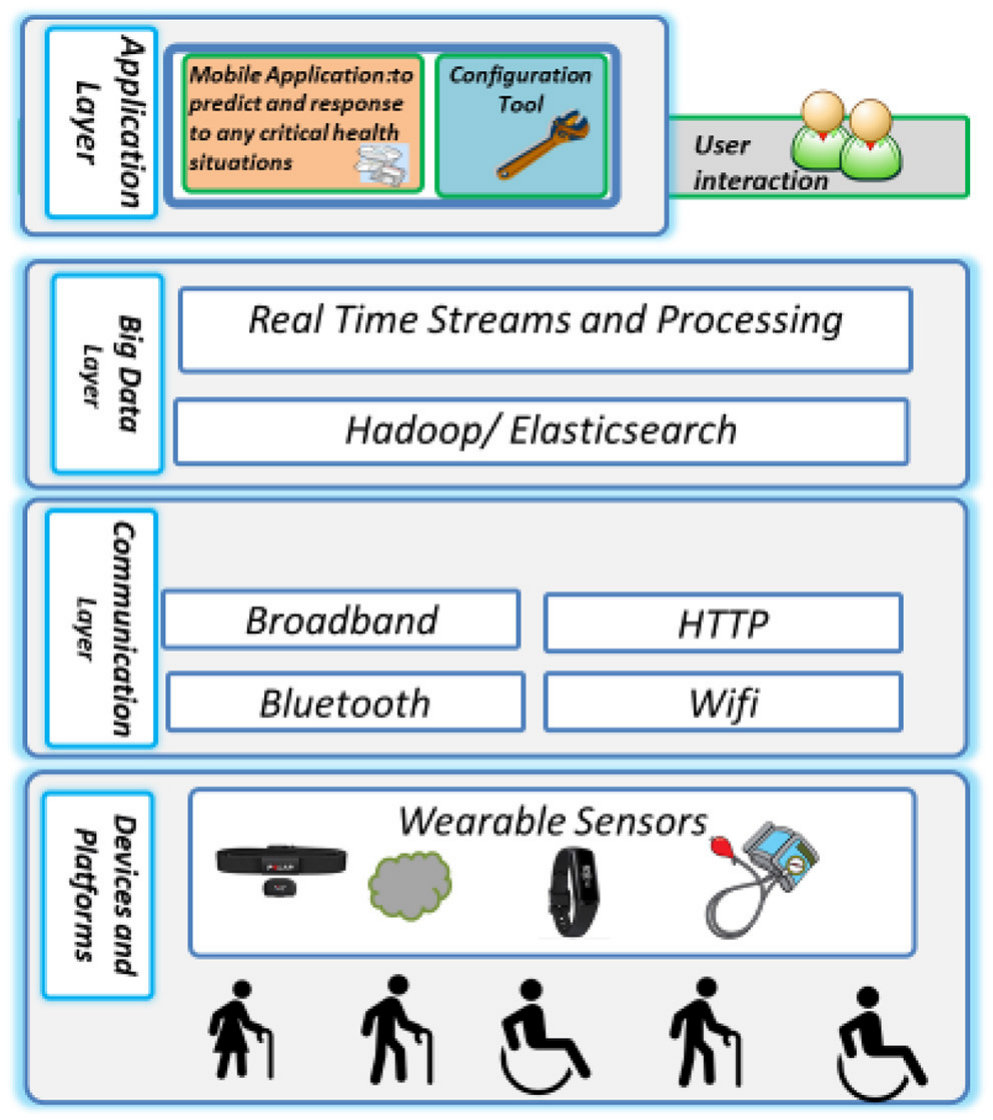
The system model.

The proposed model, shown in Figure 4, would consider heterogeneous underlying sensors and network enabling technologies for convenient, near real-time network access to status information about the health conditions resources (e.g., heart rate, blood pressure) that can be monitored and controlled with minimal management efforts or doctors' interactions. The proposed system model in Figure 4 depicts the main four layers of the proposed model, namely the devices and platforms, communication layer, Bigdata platform, and the applications layer.

- The top layer: Here resides the User applications, data analytics, dashboards used to monitor and optimize the health-related operations.
- The big data platform: This component includes big data and the corresponding standard big data platform libraries that are suitable for addressing essential needs for handling various significant amounts of data, such as Hadoop/Elasticsearch, big data components for data storage, retrieval, organization, and analysis, as well as querying big data using Hadoop. Large-scale data analysis techniques, data storage platforms, data representations, and heterogeneous data models are all part of the project. The platform also contains the libraries and functions required by the hosting system to execute local, basic, and rapid processing and filtering on data generated or received locally.
- Communications layer: This layer offers near real-time connectivity and enables the communication between Devices and Platforms such as sensors to mobile, a sensor to the sensor, and mobile to a server within the ecosystem. It includes the networking protocols required to transfer the digital information from the sensors' layer to the application layers. Heterogeneous communication technologies exist from Wireless Fidelity (Wi-Fi), Bluetooth, Wireless sensor networks.
- Devices and platforms: The foundation layer of the wearable and infrastructure layer includes system apparatus components such as mobile and server platforms. Typically, a large volume of sensor devices is used to capture the status information about the patients' health condition to translate this information to the digital world notwithstanding the fact that a wide array of sensors devices is required to collect data about the patients. The main goal of the mobile platform is to aggregate the information collected by the sensors, mobile support heterogeneous data, and communications standards. The servers host the user's applications data repositories and provide unified access to Application Programming Interfaces (APIs) for other systems and users.

#### Work Plan

- A set of initial proofs of concept centered around selected use-case scenarios illustrating the wealth of applications and technologies brought together by the researchers.
- A sub-set of mid-term partially integrated demonstrations showing the initial results of the technical achievements obtained.
- A final demonstrator integrating the results of the project as a whole and show-casing the potential for large-scale deployment for real-world field trials with application scenarios.

#### Expected Outcomes

- Integrated platforms with mobile applications with basic decision-making algorithms with limited ability to predict and respond to any critical health situations; automatic monitoring can alert doctors and patients themselves of unexpected health conditions.
- Proof of concept based on the overall infrastructure utilizing all previously described components: personal sensor network, Mobile App, and Big Data Platform melting them to one comprehensive demonstration testbed.

### Objective-3 Is Proposed to Be Achieved as Below

The aim is to develop algorithms that model and predict patients' health conditions. In the proposed platform, all smart devices ranging from low-end sensors mobile handhelds up to high-end service architectures—will interact with each other, and while doing so, they generate a stream of events in the network. Complex event processing and pattern recognition techniques will extract meaningful information for anticipating critical health conditions. By appropriately modeling event hierarchies and detecting causality between events, we will be able to transform simple events into high-level health conditions. The model must incorporate two aspects: (1) modeling component from a historical health dataset that includes publicly available health conditions with the corresponding decision making and predictions and (2) The real-time data streams generated by the wearable devices like blood pressure and heart rate etc., to forecast and predict the patient's current health conditions. The algorithm will exploit static information stored in the big data platform and in the mobile, as well as dynamic information acquired on the fly such as heart rate, activity tracking, etc. A prediction model can have three major components: (1) Target or outcome data: Data about the outcome that we want to predict, for instance, mortality risk; (2) Predictor data: Data used to make a prediction, for instance, patient symptoms, age, demographics, clinical history etc.; (3) ML model: A mathematical function that maps the relationship between the predictor data and the outcome data, for instance, decision trees, neural networks etc.

**Figure 5:**
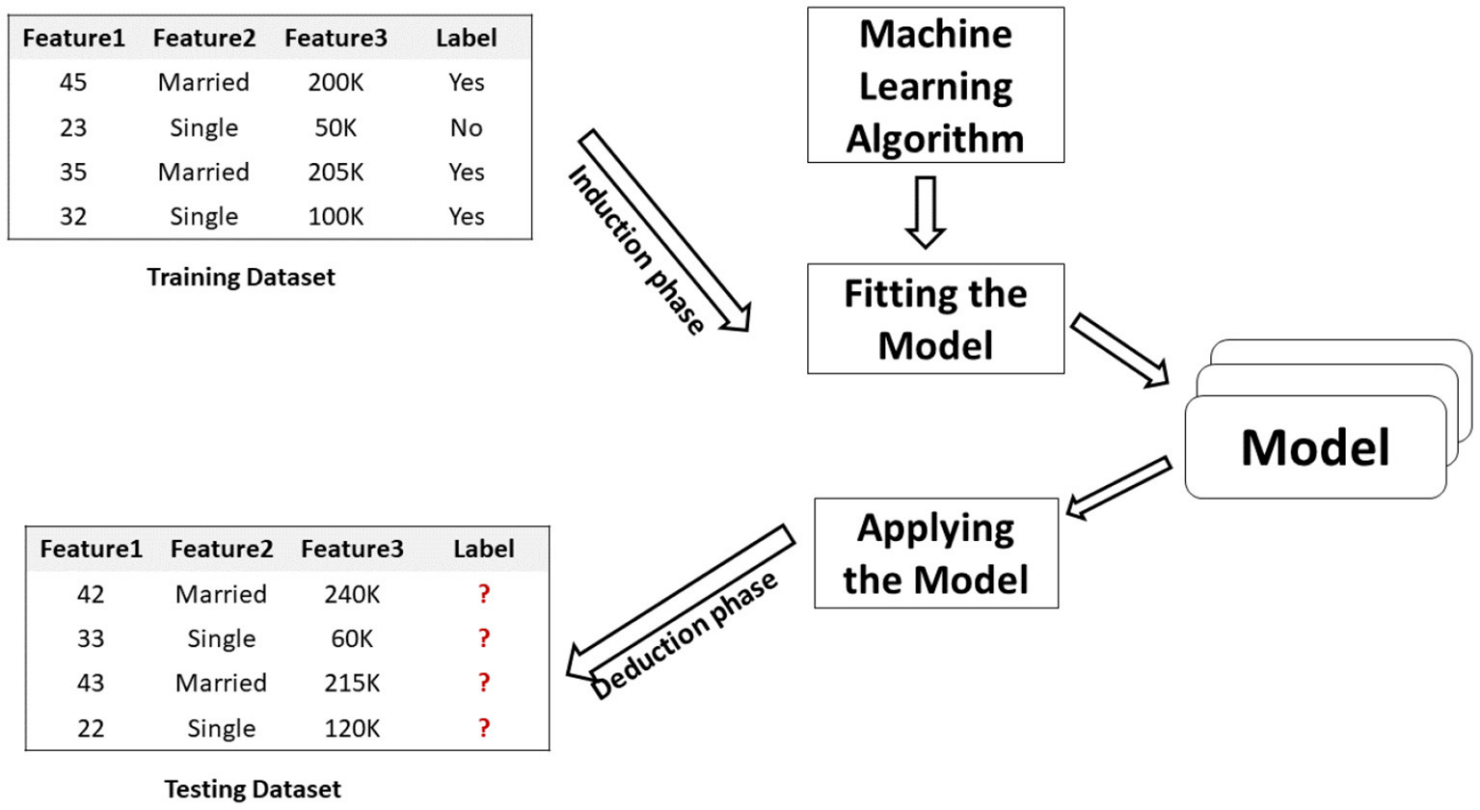
ML application.

Figure 5 depicts the typical pipeline of an ML application, starting from the input training data set and ending with the corresponding output represented by the model. The model will follow the CRISP-DM lifecycle during the proposed conceptual framework's implementation, validation, and deployment.^3^

While evaluating the ML models, the appropriate explainable AI methods will be leveraged. Explainable AI for the health care sector plays an important role in the proposed framework that helps understand and interpret predictions to the system end-user, patients and doctors.

#### Work Plan

- Investigate proper statistical and inferential representations for patients' health conditions variables that incorporate both knowledge information that is mainly edited by the researcher himself, and health variables information that is automatically generated through the proposed platform. For example, real-time health condition data and health trajectories.
- Identify appropriate learning and data mining techniques to detect anomalies in the data stream and their patient's activity—for example, machine learning methods such as Bayesian network and deep learning techniques.
- Integrate the discovered data models into the proposed platform.

#### Expected Outcomes

- Data-mining learning algorithms and information that could be used by decision-making algorithms with limited ability to predict and respond to any critical health situations.
- Proof of concept of the model integration in the platform.

### Objective-4 Is Proposed to Be Achieved as Below

This objective aims to develop and execute an effective exploitation plan for clinical validity of the holistic platform and its application.

#### Work Plan

- Preparation of an initial exploitation plan and maintenance of the plan including the identification of relevant results and the corresponding target groups.
- Involvement of the external user in the planned demonstration phase.
- Compare clinical data collected and generated by the platform with clinical information from other sources to ensure the validity of the approach.

#### Expected Outcomes

- Exploitation and use plan.
- Joint proofs of concept with external user and health data sources provider.

### Stakeholder Analysis Matrix

Table 2 Provides an initial list of the stakeholders and their expectations of the system.

**Table 2:** Stakeholder analysis matrix.

**Stakeholder name**	**Contact person**	**Impact**	**Influence**	**What is important to the stakeholder?**	**How could the stakeholder contribute to the project?**	**How could the stakeholder block the project?**	**Strategy for engaging the stakeholder**
End-Users: Patient with multi-morbidity	Phone, email address	High	High	Interact with the system on a day-to-day basis.	Agree for user interface design and functionalities to implement the new system	Reluctant to use the systems	Weekly round-table discussions
Health care authorities	Phone, email, website, address	High	High	Monitoring health conditions of the patients	Integration with the healthcare systems	Refuse to integrate new system with the health care system	Monthly round-table discussions
Doctors and health officials in case of emergencies.	Phone, email address	High	High	Monitoring health conditions of the patients	Identify system feature and accepted criteria	Reluctant to use the systems	Monthly round-table discussions
Application integrators	Phone, email address	High	High	Develop, test, integrate maintain functional system	Support the life cycle of the system		Daily round-table discussions

## Privacy and Security of the Proposed Model

As the proposed system contains highly sensitive scientific information, the system and data are prone to various levels of attacks. Following precautions that will be taken to fortify the privacy and security of the proposed conceptual framework

- **Information theft**—A suitable firewall will be installed on the servers to detect and protect from them malicious attacks.
- **DDoS attacks**—Several honey pots will be deployed to drive away attackers from the main data.
- **Ransomware**—All data stored on the server will be encrypted. Multiple copies of the sensitive and mission-critical information are backed up timely. A policy to use strong passwords and two-factor authentication with access tokens will be enabled.
- **Access control**—Different access control policies will be implemented.

## Innovations in This Research

Following are a few of the innovations from this research concept:

a. A software application and the back-end big data processing platform capable of autonomously monitoring the aged patients' health conditions in real-time serves to complement the elder's limited ability to anticipate and react to any critical health situations.
b. A truly consolidated software solution that enables autonomously monitoring the patients' health conditions in real-time serves the purpose of complementing the elder's limited ability to anticipate and react to any critical health situations.
c. Modern data science application has helped many patients decrease or sometimes even eradicate health condition uncertainty. It has helped doctors to harness information and bring an insight that might be useful for complex treatment progress such as cancer treatment.

Therefore, the proposed concept has the potential to create a whole new business ecosystem and exert social influence that may go way beyond the current health care system as we know it today. Both aspects are crucial for the acceptance and the wide deployment of such a system. Therefore, there is high potential for emerging new business opportunities and business models, which might benefit small and medium enterprises (SMEs) and the industry serving health care providers.

The project will be led by Dr. Shadi Atalla and supported by Dr. Saad Ali Amin and Dr. Manoj Kumar M V from Nitte Meenakshi Institute of Management (NMIT)- Bangalore, Dr. Nanda Kumar- from Ramaiah Medical College-Bangalore. Prof. Wathiq Mansoor will oversee the technical aspect of the project, while Prof. Ananth Rao will monitor and evaluate the project work plan and outcomes for timely completion of the project with financial assistance from local partners. NMIT, through Bangalore Ecosystem for Health Research (BEHR) network, would provide expert advice to the administrative core, data management core, and research projects on topics including data management; data analysis, including machine learning and artificial intelligence; and data visualization through its Data Management Analysis Core (DMAC) project.

## Sustainability

Sustainable practices improve the delivery of health care services in a variety of ways, from lowering the environmental impact of facilities to leading efforts to address public health hazards posed by climate change. Adopting sustainable practices in health care has the greatest impact by addressing the environmental and social determinants of health by building healthy living and working environments. The proposed conceptual model autonomous tool for Monitoring Multi-Morbidity Health Conditions in UAE and India will take almost every step to efficiently handle any sustainability-related issues right from the day one of implementation.

## Data Science Technology That Is Being Adopted

Modern data science application has helped many patients decrease or sometimes even eradicate health condition uncertainty. It has helped doctors to harness information and bring an insight that might be useful for complex treatment progress such as cancer treatment. The project team would rely extensively on leveraging their experience of applying data mining and ML technologies to different scenarios ranging from data gathering, cleaning, processing, visualization, management, modeling, and finally producing reproducible data science products for building health care intelligent recommendations based on patient clustering and profiling. The project will use big data and the corresponding standard big data platform to address substantive needs for handling various significant amounts of data, such as Hadoop/Elasticsearch, big data components for data organization, storage, retrieval, and analysis, as well as querying big data with Hadoop. Large-scale data analysis, data storage systems, data representations, and semi-structured data models are all part of the project.

## Data Sets

There are many public repositories of data sets that contain health data sets. These data sets are typically public and open access and allow for testing algorithms very quickly. Such as Kaggle, UCI Machine Learning Repository, Big Cities Health Inventory Data, Healthcare Cost and Utilization Project (HCUP), data.gov, Kent Ridge Bio-medical Dataset, HealthData.gov, MHEALTH Dataset Data Set, and TILDA dataset. Notwithstanding, the validation of the phase of the project will rely intensively on simulated health data. These data will be gathered using commodity devices used by the researchers themselves. The system would be built and evaluated as part of a Proof of Concept Trial, which would take place at a trial site in Dubai. The system would be tested by the researchers to gain confidence in the prototype. Later the researchers can also include members of their network for pilot testing before large-scale validation in Dubai and India-Bangalore. Hence, public healthcare datasets would become essential in Dubai (available through our network), and India (publicly available through the Ministry of Health and Family Welfare database). These would be used to build Machine Learning Prediction Models.

## Data Governance

The project team would encourage industry cooperation to harness current technology and cost-effective and sustainable solutions. The following SMART goals will be strictly implemented and made compliant with.

- Service cost model that is both sustainable and transparent.
- Follow worldwide authentication policies and services.
- Observe international data protection and anonymization standards.
- Metrics for performance and consumption will be measured and shared.
- Periodical data integrity check and reporting.
- Carry out risk-based validation.

## Application Programming Interfaces and Integration With Legacy Systems

The proposed conceptual framework provides a user workspace to store, manage, compute, and share the user's data and analysis results with collaborators or the larger research community. Further, the architecture does provide a platform wherein users and researchers can also propose ideas or implementations that can be incorporated upon following the proper guidelines and policies of the proposed tool. It allows data to be combined across users and researchers in conjunction with other data openly accessible via the proposed tool. Researchers are also provided with open data APIs by the proposed tool, making the data accessible, understandable, and actionable, tailored to the unique needs of the authorized users or valid researchers.

Through multiple open-data APIs that may be made available to various collaborators utilizing portable Apps (web/mobile), the proposed tool enables portable tools to be deployed for one's personal usage, shared with collaborators, or free with the larger research community. The planned work is available under an open-source license and can be accessed *via* GIT, GITHUB, or other commercial platforms.

The proposed tool caters to a wide range of user personas, including novices and computationally sophisticated users, by offering a web interface as well as API access to data, tools, and computation, as well as facilitating integration with other systems. This is achieved by the proposed architecture's provision of resources that form the web portal via which registered users/researchers may have secure access to the data. The online portal will also feature usage guidelines and a few sample experiments for novice and experienced users to familiarize themselves with the site.

## Expected Results

The project would provide an innovative integrated software solution for monitoring elderly persons experiencing multi-morbid health conditions. To achieve this result, several aspects of the system that spans from algorithms and mobile app to platforms would be closely investigated and continuously improved with innovations. The following list provides a short description of the most relevant results expected at the end of the project:

- The project would redefine application requirements and technical specifications to develop the described integrated software solution.
- The project would develop lightweight data communication mechanisms, protocols, and cryptography for privacy protection that are computationally inexpensive and require minimum bandwidth, implementable in simple devices, and flexible for distributed approaches.
- The project would provide a generic information model representing various system parameters such as network, software, security, and performance parameters data management operations.
- The project would provide a prototype of an autonomic, generic, and extensible execution platform that will allow smooth interactions between patients and its mobile application and the back-end big data platform.
- The project would improve the accuracy of the data mining predictive model to identify patients with critical health conditions.

## Commercialization of Research

As discussed in the foregoing sections, there exists a great potential for creating business opportunities and business models to benefit SMEs as well as the industry acting as health care providers. This ensures the scope for commercialization of the research concept and prototype in due course.

## Ethical Issues

Although the project is focusing on the technical and implementation issues from big data platform and solutions perspectives, we cannot overlook the issues created by its possible global implementations such as ethics, policies, and legal issues that face computer professionals and data scientists while working with health and personal datasets, as well as the project, will examine the related cyber security issues.

## Legal Issues

The project will maintain best practices and IEEE guidelines Intellectual Property, privacy issues, laws, and professional ethics governing these issues while working with the systems. The legal responsibilities need to be clearly defined in cases of system down or malfunctions by introducing new laws and regulations to handle and manage such cases.

## Cultural and Social Implication Issues

The system would introduce a significant social implication by changing the new way of interaction between the patients and their doctors, and its effect on the medical work lifestyle as with the new application, the doctor will always be aware of the patient's current health condition any time, and anywhere.

## Proposed Research Milestones and Gantt Chart

**Figure 6:**
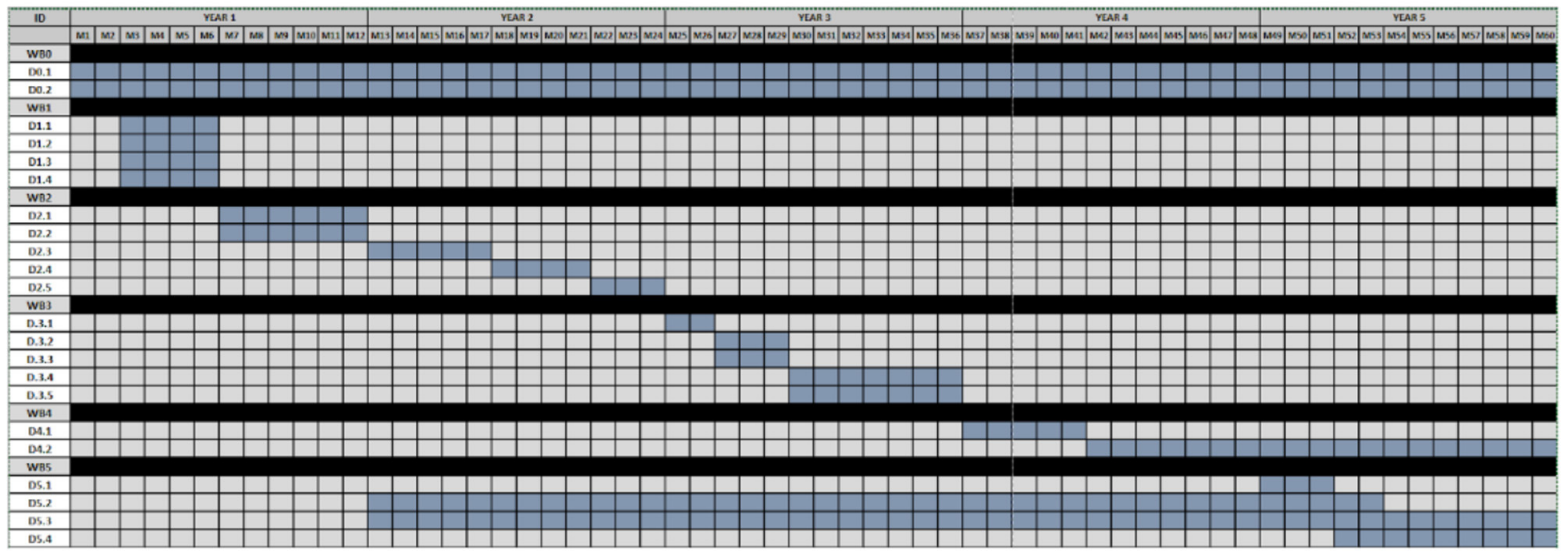
Gantt chart of project milestones.

The proposed Project package breakdown structure and Gantt chart are shown in Figure 6, Tables 3 and 4.

**Table 3:** Proposed project yearly plan.

**Year of award**	**Months**	**Research activity milestones (team of 5 researchers formed already + 2 young researchers)**
1st year	1–2	1. A team of 5 members will refine the research proposal; allocate responsibilities for each member, one to organize data, 3 to do literature review; 5th to organize the delivery of Objective 1
		2.Two young researchers with MS and fresh PhDs to train them in problem identification, literature review, hypothesis setting, Data Science tools being used, the process of Platform development.
	3–6	3.Define the application use cases and requirements
		4. A defined subset of use cases of the solution.
		5. Complete literature review
		6. Refine framework for the platform
	7–12	7. Develop the mobile application with basic decision-making algorithms.
		8. Integrate the Mobile App with described components: personal sensor network, medical devices, and wearables.
2nd year	1–4	1.Young researchers and the team (TEAM) to prepare draft paper for internal presentation in a research forum. Obtain feedback from forum peers.
		2.Revise the draft with the feedback
		3.Complete logistics for presenting the paper in 1st International Symposium.
		4.Refine framework for the Big Data platform
	5–8	5. Present the paper in the Bangalore Ecosystem for Health Research (BEHR) symposium
		6. Obtain feedback from symposium participants
		7. Incorporate feedback to publish the symposium proceedings
		8. Disseminate the research results technically to the local UAE and Indian community through social media channels.
		9. Integrate the Mobile App with the BigData Platform
	9–12	10. Submit the proceedings to a peer-reviewed journal
		11. Develop proof of concept based on the overall Solution infrastructure
		12. Refine the AT tools for commercial.
3rd year	1–2	1. A team of 5 members will refine objective 3 of the research proposal; allocate responsibilities for each member, one to organize data, 2 to do literature review; 4th to organize the delivery of Objective 3 and 4
		2.2 young researchers to train them in problem identification, literature review, hypothesis setting, Machine learning tools being used, process of Autonomous Tool (AT) integration the ML models with the solution platform.
	3–6	3. Complete collection of existing publicly available and/or UAE and Indian data.
		4. Refine the literature review with a focus on machine learning.
		5. Refine framework for the AT for the integration ML model.
		6. Set Hypotheses and goals.
	7–12	7. Integrate the AT
		8. Run experiments with in-sample data
		9. Validate the AT using test data or validation data
		10. Start preliminary analysis on initial results
		11. Apply AT on the population: researchers themselves and their relatives
		12. Get feedback from the population on the merit and demit of using the AT for improving their health.
		13. Use the feedback as additional data and feed to the AT tools
		14. See the improvements in the results
		15. Iterate the cycle 11–14 three times to cover at least the sample number of patients in the local population to get a decent response.
		13. Conduct community forums to disseminate the iterated results
4th year	1–4	1.Young researchers and the team (TEAM) to prepare draft of the second paper for internal presentation in a research forum. Obtain feedback from forum peers.
		2. Revise the draft with the feedback
		3. Complete logistics for presenting the paper in 2nd DS- I UAE and India Symposium
	5–8	4. Present the paper in the Bangalore Ecosystem for Health Research (BEHR) symposium
		5. Obtain feedback from symposium participants
	9–12	6. Incorporate feedback to publish the symposium proceedings
		7. Submit the proceedings to a peer-reviewed journal
5th year	1–4	1. The Team members will refine objective 4 of the research proposal; allocate responsibilities for each member, one to commercialize the tool by patenting its data, 2 to Disseminate in non-technical; 2 to organize the delivery of the exploitation plan
	5–8	2. Disseminate in a non-technical manner the research results to the local UAE and Indian community through social media channels
		3. Preparation of an initial exploitation plan and maintenance of the plan including the identification of relevant results and the corresponding target groups.
		Involvement of the external user in the planned demonstration phase
	9–12	4. Compare Clinical data collected and generated by the platform with clinical from other sources to ensure the validity of the approach
		5. Joined proofs of concept with external user and health data sources provider.
		6. Closure of the research project with a summary of lessons learned and experiences gained by the TEAM for future

*WB, Work Breakdown; Description: (M—Month; PM—Project Month)*.

**Table 4:** Project work breakdown structure.

**WB**	**Work breakdown title**	**PM**	**Start**	**End**
**WB0**	**Project management**	5	M1	M60
D0.1	Administrative project management	5	M1	M60
D0.2	Impact management	5	M1	M60
**WB1**	Cases requirements gathering and literature review			
D1.1	Use cases and user requirements	7	M3	M6
D1.2	Use cases of the proposed solution	7	M3	M6
D1.3	Objective 1 and 2 literature review	4	M3	M6
D1.4	Refine framework for the platform	4	M3	M6
**WB2**	Platform development and implementation			
D2.1	Mobile application with basic decision-making algorithms.	5	M7	M12
D2.2	Integrate the Mobile App with medical devices, and wearables	5	M7	M12
D2.3	Refine framework for The BigData Platform	5	M13	M17
D2.4	Integrate the Mobile App with the BigData Platform	5	M18	M21
D2.5	Develop proof of concept based on the overall solution infrastructure	5	M22	M24
**WB3**	Machine Learning and Autonomous Tool (AT) integration			
D3.1	Literature review with focus on machine learning	7	M23	M24
D3.2	Data identification and collection	7	M25	M28
D3.3	Refine framework for the AT for the integration ML model	7	M25	M28
D3.4	Set hypotheses and goals	7	M29	M36
D3.5	Building data models	7	M29	M36
**WB4**	Integration and testing			
D4.1	Integrate the Machine learning Model with the Platform	5	M37	M40
D4.2	Run testing scenarios	7	M41	M60
**WB5**	Evolution, dissemination evolution exploitation			
D5.1	Preparation of an initial exploitation plan and maintenance	7	M49	M51
D5.2	Platform evaluation and validation		M13	M56
D5.3	Publication and commercialization	7	M13	M60
D5.4	Disseminate in non-technical manner	7	M52	M60

## Conclusion

The proposed research concept aims at integrating several real-time monitoring technologies and data science methods to promote a safe and independent living of the elderly multi-morbidity patients through health data sharing and communication between patients and healthcare professionals. The solution relies on the interaction of the elderly persons with multi-morbidity diseases with commodity devices to enhance the elder's limited ability to watch, predict and respond to any critical health situation. In conclusion, autonomous observance of the patient's conditions along with vigilant doctors and patients themselves will help to minimize the unexpected health complications.

## Author Contributions

All authors listed have made a substantial, direct, and intellectual contribution to the work and approved it for publication.

## Conflict of Interest

The authors declare that the research was conducted in the absence of any commercial or financial relationships that could be construed as a potential conflict of interest.

## Publisher's Note

All claims expressed in this article are solely those of the authors and do not necessarily represent those of their affiliated organizations, or those of the publisher, the editors and the reviewers. Any product that may be evaluated in this article, or claim that may be made by its manufacturer, is not guaranteed or endorsed by the publisher.

## Footnotes

^1^Ericsson Consumer lab Analytical platform 2010-2020.

^2^The proposed conceptual technique featured as natural is based on the fact that the majority of the healthcare systems are migrating to ICT-enabled workstyle. Unfortunately monitoring, multi-morbidity health conditions have been overlooked by current research and development. It is naturally required to migrate this system to ICT-enabled facility, and this paper proposes the means to achieve it.

^3^The CRoss Industry Standard Process for Data Mining (CRISP-DM) is a process model with six phases (business understanding, data understanding, data preparation, modeling, evaluation and deployment), that naturally describes the data science life cycle.
